# Parental exhaustion during COVID-19 pandemic: links to relationship outcomes and dyadic coping

**DOI:** 10.1007/s12144-023-04658-2

**Published:** 2023-05-16

**Authors:** Matilde Carvalho, Marisa Matias

**Affiliations:** 1 Faculty of Psychology and Educational Sciences, University of Porto, Porto, Portugal; 2 Faculty of Psychology and Educational Sciences, University of Porto Center for Psychology at the University of Porto, Porto, Portugal; 3 R. Alfredo Allen, Porto, 4200-135 Portugal

**Keywords:** Lockdown, Parental exhaustion, Relationship satisfaction, Conflict frequency, Dyadic coping

## Abstract

The COVID-19 pandemic and lockdown altered families? routines, from telework imposition to performing additional childcare, as children started studying at home. Adapting to these demands can have effects on couples’ relationships. This study aimed to analyze couples? experiences of parental exhaustion during the lockdown and to understand its links to relationship satisfaction and conflict frequency. It also explored how couples’ internal resources (e.g., dyadic coping) buffered these effects. We examined data from 210 individuals in a romantic relationship who were cohabiting with their partners, teleworking, and had children under 18. Absolute values of parental exhaustion and relationship quality were not severe, but there was evidence that parental exhaustion contributed to decreasing relationship satisfaction and increasing conflict. Positive forms of dyadic coping were found to moderate only the adverse effects on conflict frequency. Implications of these results to couple’s support under stressful events are provided.

## Introduction

The COVID-19 pandemic has undeniably impacted the lives of many individuals and families. The first positive cases of the novel coronavirus occurred in Portugal in March 2020, so a mandatory lockdown was implemented for the entire population. Teleworking became obligatory as often as possible, and students were sent back home for online schooling. According to national data, by the end of May 2020, half of the Portuguese companies were using telework. On the last day of the month, there was a total of unprecedented 14,094 online lectures given (Fundação Francisco Manuel dos Santos, 2020). As children stayed home for school, demands for extra support increased while parents had to manage their own work from home. This may have impacted the romantic relationship of these parents. This study describes how these parents’ romantic relationships, namely relationship satisfaction and conflict, were affected by the constraints brought up by the pandemic and if resources such as dyadic coping were used to ameliorate parents’ romantic relationship well-being.

### Relationships during COVID-19

Explored by Ward and collaborators (2009), relationship satisfaction within couples can be defined as an emotional state in which individuals are admittedly satisfied with interactions, experiences, and expectations in terms of the couple’s life and is considered a valuable measure of its quality as it provides a subjective perspective about the partner and the relationship itself, framing the relationship quality with its involving phenomena (Hendrick et al., 1998). Dealing with stress as a couple can sometimes lead to conflicts associated with negative perceptions of the relationship, like low satisfaction and more considerable instability as a cause and a consequence across time (Johnson et al., 2018). External stress has a negative impact on the quality and satisfaction of relationships and can be considered a predictor of poorer communication that can lead to separation (Bodenmann, 2006). Thus, understanding the perceptions of each individual about their relationship and assessing the degree of conflict may allow for an in-depth view of relationships’ dynamics and quality.

The COVID-19 pandemic brought different forms of stress into the family context, especially for couples living together. Ogan and collaborators (2021) examined how three types of stress associated with the pandemic were related to changes in romantic relationships across six months. Authors found that global perceived stress, not economic pressure or pandemic concerns, was associated with increased relationship instability. A greater sense of loneliness, financial strain, and stress due to the pandemic was also associated with lower relationship satisfaction and more conflicts within marital relationships (Balzarini et al., 2020). The pandemic led to families having to discuss topics of conversation they never had dealt with before, which was associated with perceived harm in their relationship closeness (Johnson et al., 2021). Across the lockdown period, couples reported more disagreements than usual and more verbal fights (Lee et al., 2021) related to changes due to the pandemic (Balzarini et al., 2020; Luetke et al., 2020). Nevertheless, the pandemic brought positive and negative communication changes, with increased arguments and understanding (Vowels et al., 2021). Despite spending more time together, the disposition for intimacy also decreased during these times, as partners encountering COVID-19 related conflicts were found to have a lower frequency of intimate and sexual behaviors than those not experiencing any such conflict (Luetke et al., 2020). Couples showed decreased intimacy behaviors, describing lower sexual desire, lower sexual intercourse frequency, and other sexual-related activities, which was directly associated with the quality of the partners’ relationship (Li et al., 2020). It can be stated that the pandemic reduced the quality of romantic relationships in its different dimensions, having increased stress factors towards couples which created opportunities for more conflicts and may have led to a perception of less satisfaction with the relationship.

### Parental exhaustion during COVID-19

As individuals and parents had to stay at home full time, some already existing conflicts when it comes to managing different life domains were brought to life. When part of a family and working from home, one can be involved in more than one life sphere at the same time, which can result in a better job-related performance and decrease family life satisfaction (Peng et al., 2011). This blurring on work and family roles has been found to have a negative impact on family life, and more specifically, on relationship satisfaction, as individuals who blend roles in one sphere tend to do it in other areas as well, resulting in poor psychological well-being and consequently poorer satisfaction with the relationship with the partner (Paulin et al., 2017). Furthermore, the instability of work schedules can also have an impact on the quality of family and marital relations, as higher job-related constraints and frustration can contaminate the relationship with the partner. Mills and Täht (2010) found not only that this kind of pressure had an influence on relationship satisfaction but also that gender-related differences can be identified. This effect was positive for men, as more pressure from job increases their levels of satisfaction while the relationship is negative for women. The experience of the COVID-19 lockdown was a significant and continuous exposure to this blurring of boundaries, as families and specifically couples had to adapt to a complete change in their habits (Jacukowicz & Merecz-Kot, 2020), spending more time together but also shifting their routines and plans to manage working and parenting responsibilities (Vowels et al., 2021). Nevertheless, COVID-19-associated lockdown measures were found to have both positive and negative effects on family life, on the one hand, leading to a shared and more balanced distribution of tasks related to housework and childcare (Carlson et al., 2022) and, on the other, to an increase in domestic demands (Craig & Churchill, 2021).

When it comes to the challenges associated with parenting responsibilities there are a lot of factors to be considered such as parents’ individual skills, children characteristics and type of interactions and the cooperation between the two partners (Berryhill et al., 2016; Khajehei, 2016; Mikolajczak et al., 2018). These demands can be associated with the level of relationship satisfaction with the partner, as the quality of the relationship can determine the parental experience and the last can influence the first (Khajehei, 2016). Lavee and collaborators (1996) explored in depth these associations and described how parental-related stress can affect the quality of the parents’ romantic relationship. According to their findings, partners influence each other so much that one partner’s stress associated with the parental role can have an impact on the other partner’s feelings and behaviors. Indeed, in a study following families over five years, Berryhill and colleagues (2016) found that parents who had problems dealing with their children in an early stage of their development showed parental stress a few years later and lower quality and satisfaction with the relationship with their partners as the years went by. Significant and chronic exposure to this kind of stress can sometimes escalate into parental burnout which comprises three dimensions: an overwhelming feeling of parental related exhaustion, an emotional distancing from their children and an inefficacy feeling towards the parental role (Mikolajczak et al., 2018). The number of children and their age, having a special needs child, and being a single parent has been found to enhance the risk of parental exhaustion (Marchetti et al., 2020) and in addition to these, socioeconomic level and the variety of each partner’s roles also contributed to its association with relationship quality (Lavee et al., 1996).

During the COVID-19 pandemic, parenting demands were enlarged due to more time spent taking care of the children, fewer resources in childcare, and more variety of children’s needs (Ares et al., 2021; Griffith, 2020), alterations in children’s behaviors (Mantovani et al., 2021), making efforts to maintain routines and finding creative ways of entertaining their children and in addition having to adapt to working from home at the same time (Coyne et al., 2020). All these factors contributed to increased parental exhaustion (Marchetti et al., 2020), present both in mothers and fathers (Aguiar et al., 2021). Throughout the lockdown period, parental burnout, less satisfaction with the parental role, and unhelpful coping strategies used by the parents contributed and were found as factors to increasing parental burnout levels (Skjerdingstad et al., 2021). Indeed, as previously stated by Mikolajczak and collaborators (2018), coparenting dynamics and family functioning explained parental exhaustion more than children’s characteristics or sociodemographic factors, reinforcing how heavier forms of this exhaustion can be avoided if better types of communication between the parents and more balanced management of childcare tasks are implemented.

### The role of dyadic coping

While being part of a relationship, partners can face stress individually and conjointly. Suppose one individual is incapable of regulating stress alone or their efforts go against the partner’s expectations. In that case, it often spills into the relationship, and the problem becomes a problem of the two (Bodenmann, 1995, 1997). In its Systemic-Transactional Model, Bodenmann (1995, 1997) stated that the experience of dyadic stress includes not only the consequences of this stress on the relationship but also the conjoint efforts to evaluate it by assessing its significance and demands for the couple and what resources they have to cope with. Dyadic coping is defined as the process of taking into consideration stress signals from both partners and their coping reactions to one another and therefore can be framed by the source of the stress, the dynamic interactions between both partners, and the strategies and aims implemented individually and jointly (Bodenmann, 1997). According to the same author, different types of dyadic coping can be described, considering the type of situation the couple faces and the individual and dyadic assessment. Following this Systemic-Transactional Model (Bodenmann, 1995, 1997), dyadic coping can be problem-centered, taking form in practical actions performed by the partners such as equal division of tasks, or emotion-centered, for instance providing emotional support to the partner. It can also be negative or positive, by helping the partner unwillingly and in a hostile way, or on the other hand, by assisting the partner with solidarity and developing conjoint actions to solve the problem.

Dyadic coping has been shown to have a positive effect on the quality of romantic relationships, resulting in better communication, well-being, and fewer psychological problems (Bodenmann, 2005, 2006). This conjoint way of facing stress is more performed by happy and stable couples than unhappy and unstable ones. It is a moderator of the negative association between stress and relationship quality (Bodenmann, 2005, 2006; Falconier et al., 2015). Bodenmann (1995, 1997) specifies three types of positive dyadic coping, including a common form where both partners participate in the coping process actively, a supportive form where one partner helps the other who is less prepared to handle the problem, and a delegated type of dyadic coping, where one partner takes over the other’s duties. These positive forms of dyadic coping can be a useful resource in romantic relationships and are associated with higher quality of romantic and sexual life, more constructive conflicts and less destructive ones (Falconier et al., 2015), feelings of sharing, and high satisfaction with the relationship (Vedes et al., 2013) and an increase in the perception of unity with the partner rather than two independent units (Vedes et al., 2015).

During COVID-19, despite couples reporting lower quality of the marital relationship, a buffering effect on this relationship was found for those who perceived responsiveness on the partner (Balzarini et al., 2020). Couples who entered the lockdown period and faced its adversities with pre-existing better communication and support strategies were expected to maintain high relationship quality; more than those who did not adopt these dyadic processes (Pietromonaco & Overall, 2020). As so, the perception of support from the partner seems to be an important factor in diminishing the spillover of external stressors like the ones brought by COVID-19-related constrains into the romantic relationship. These findings are in line with Vowels and colleagues’ (2021) investigation, which revealed that couples identified conjoint coping techniques towards lockdown demands that helped them protect their relationships, like increasing the sharing of housework and childcare chores. Indeed, couples who reported higher levels of dyadic coping revealed fewer disagreements and fights during the COVID-19 pandemic; however, when analyzed as a moderator, dyadic coping did not buffer individuals from increases in relationship conflict (Lee et al., 2021). Moreover, Ogan and collaborators (2021), showed that dyadic coping was not a mediator of the links between perceived stress during the lockdown and relationship stability. Both studies recognize the inconsistency of these findings with previous literature about dyadic coping’s moderator effects. Nevertheless, they still suggest that dyadic coping can be a source of protection for relationships even when stress factors are controlled.

### The current study

Although COVID-19 related stress has been found to have adverse effects on individuals and couples, and dyadic coping seems protective against these stressors, there is not, to our knowledge, any research associating specifically parenting exhaustion during the pandemic with the quality of romantic relationships for cohabiting couples and dyadic coping. There is also no moderating evidence of dyadic coping as a buffer to all these constraints of the relationship.

With this study we intended to contribute to the characterization of how couples experienced the early stages of the COVID-19 pandemic in their relationships and parenting roles. We also aimed to understand how parenting exhaustion could be associated with the quality of the relationship and to test if dyadic coping served as a moderator of these links. We hypothesized that (H1a) individuals would show low levels of relationship satisfaction and high conflict frequency overall. In addition, we hypothesized that (H1b) high levels of parental exhaustion would be found. Secondly, we predicted that (H2) parenting stress would be negatively associated with relationship satisfaction and positively associated with conflict frequency. Lastly, we hypothesized that (H3) dyadic coping would moderate the associations between parenting exhaustion with relationship satisfaction and conflict frequency.

## Method

### Procedure

Data for this study was drawn from a larger project on the experience of COVID-19 pandemic lockdown with different sections only to be responded to if criteria were met (e.g., being in a relationship, having children). In order to participate, respondents had to be older than 18 years of age. Survey participants were recruited between April and June 2020 through authors’ institution emailing lists, including all teaching, researchers, and technical staff, and also students. Social media, namely Facebook closed and open support groups directed to parents and where parenting content was continuously shared, were also used to disseminate the survey. During this time, Portugal was under severe lockdown and work restrictions, as children were sent back home to online school, and telework was mandatory as often as possible. The study was approved by the Ethics Committee of the authors’ institution, and participants had to confirm their consent to proceed with the survey.

A total of 1327 individuals completed the survey, from which only those in a romantic heterosexual and cohabitating relationship, who had children under 18 and were working from home, were selected. Participants who did not respond to any item of the study scales were also excluded, and multiple imputations were performed on the remaining missing answers. Using this criteria resulted in a sample of 210 individuals.

### Sample

The sample was comprised mainly of women, aged 28 to 59 years (*M* = 42.8, *SD* = 5.73). Most participants had a high education level, with 64.3% having completed at least High School or a higher level. Many individuals (58.1%) had medium household income, and for the majority (68.1%), their financial status did not change after the lockdown. Almost half of their partners also worked from home during this period (49.5%). Participants had between 1 and 4 children whose ages ranged from less than a year to 17 years old, with 70% noticing some or many alterations in the youngest child’s behavior after the lockdown. When experiencing the first months of lockdown, 45.2% of the individuals reported leaving the house only once a week, 28% would never leave or do it every two weeks, and 26.7% would go outside often in the week or every day. Additional demographic information is available in Table 1.

**Table 1:** Descriptive Statistics for Sociodemographic Variables

	Descriptive Statistics			
	Descriptive		Frequency	
Variable	Mean	SD	Counting	%
Age	42.8	5.73		
Gender ^a c^				
Men			40	19.0
Women			170	81.0
Education level (years)	17.4	2.50		
Satisfaction with current financial situation (1 = Very unsatisfied to 10 = Very satisfied)	6.21	2.13		
Professional Status				
Teleworking			202	96.2
Employed but at home in childcare			13	6.20
Working at the workplace			17	8.10
Working but not at the usual workplace			4	1.90
Layoff			3	1.40
Other			7	3.40
Working hours (a week)	36.2	15.1		
Days in telework (a week) (n = 208)	5.38	1.12		
Partner’s Professional Status				
Teleworking			104	49.5
Employed but at home in childcare			9	4.30
Working at the workplace			68	32.40
Working but not at the usual workplace			6	2.90
Layoff			15	7.10
Unemployed			11	5.20
Other			14	6.90
Relationship Duration (years)	18.4	6.75		
Cohabitation Duration (years)	14.8	6.41		
Number of children				
1			77	36.7
2			108	51.4
3			21	10.0
4			4	1.90
Youngest child’s age	7.81	4.91		

### Measures

***Relationship Satisfaction.*** As all participants were in a cohabiting relationship, but not all were married to their significant other, we resorted to the Relationship Assessment Scale (RAS: Hendrick et al., 1998). Four items of the RAS were used to measure this construct in its Portuguese version (Santos et al., 2000). A sample item is “My partner meets my needs.” (1 = *nothing*; 5 = *completely*). The scale was shown to have good internal consistency in our sample (*α* = 0.92). The satisfaction factor has been increasingly valued when assessing relationship quality, particularly in the presence of parental stress (Randall & Bodenmann, 2017) and the RAS scale has been the most commonly used when evaluating its interaction with dyadic coping (Falconier et al., 2015).

***Conflict Frequency.*** One single question was used to assess how often couples were fighting after the lockdown imposition, as did other authors when studying relationship changes during this period (Balzarini et al., 2020; Lee et al., 2021): “Comparing to the period before lockdown, how often did you and your partner have had a fight or a conflict?”. Answers were again displayed on a Likert scale ranging from 1 = *much less frequency* than before to 5 = *much higher frequency* than before.

***Parental Exhaustion.*** Roskam’s Parental Burnout Assessment scale (PBA: Roskam et al., 2018; Portuguese version: Matias et al., 2020) was used to assess parental exhaustion as in other studies focusing on the pandemic experience (Aguiar et al., 2021; Griffith, 2020; Skjerdingstad et al., 2021). The PBA comprises four subscales: Emotional Exhaustion, Contrast, Feelings of Being Fed Up, and Emotional Distancing. This study used four items of the Emotional Exhaustion subscale (e.g., “I feel completely run down by my role as a parent”). Items were rated on a 6-point frequency scale (1 = *never*; 2 = *once in the last weeks*; 3 = *once a week*; 4 = *occasionally in the week*; 5 = *daily*; 6 = *several times a day*) and also demonstrated a high internal consistency for this sample (*α* = 0.94).

***Positive Dyadic Coping.*** Dyadic coping has broadly been used in assessing couples’ dynamics and particularly facing the COVID-19 pandemic (Ogan et al., 2021). The Portuguese adaptation (Vedes et al., 2013) of the Dyadic Coping Inventory (DCI: Bodenmann, 2008) was used to measure positive dyadic coping. DCI is a 37-item questionnaire that covers both self-report and perceived partner versions of the four dimensions of dyadic coping (Supportive Dyadic Coping, Delegated Dyadic Coping, Common Dyadic Coping, and Negative Dyadic Coping) with answers falling on a 5-point scale ranging from 1 = *very rarely* to 5 = *very often*. In this study, we resort to the perceived partner version of the Supportive and Delegated Dyadic Coping comprising seven items (e.g., “My partner helps me to see stressful situations in a different light.”) which weighted result in positive dyadic coping scores. High internal consistency was once again found in this sample (*α* = 0.85).

### Covariates

Number of children and the youngest child’s age, number of days in telework, partners’ working status as being in telework (*Yes* or *No*), degree of satisfaction with the financial situation (1 = *Very unsatisfied*; 10 = *Very satisfied*) and gender were considered as covariates.

### Data analysis

Data was thoroughly analyzed using SPSS (software IBM® SPSS® v.26). Items of the RAS, PBA, and DCI were calculated by averaging individual’s item responses into one single score per participant to form total scores for relationship satisfaction, parental exhaustion, and dyadic coping, respectively. Missing data due to incomplete responses ranged from 2.8% in parental exhaustion items to 21.8% in relationship satisfaction. This data was completed through multiple imputations and pooled into one result per line. All variables had acceptable values for skewness and kurtosis, as shown in Table 2 and according to Kline (2005) (Sk < 3; Ku < 7).

**Table 2:** Correlations between Study Variables and their Descriptive Statistics

					Descriptive Statistics					
Variable	1	2	3	4	Mean	*SD*	Skewness	*SE*	Kurtosis	*SE*
1. Relationship Satisfaction	-	-	-	-	3.78	0.91	− 0.44	0.17	− 0.78	0.34
2. Conflict Frequency	− 0.30***	-	-	-	2.90	0.88	0.07	0.17	0.56	0.33
3. Parental Exhaustion	− 0.32***	0.33***	-	-	2.63	1.44	0.56	0.17	− 0.75	0.34
4. Dyadic Coping	0.74***	− 0.23**	− 0.24***	-	3.47	0.79	− 0.41	0.17	0.04	0.34

**Note**. * *p* < .05; ** *p* < .01; *** *p* < .001

Bivariate correlations were performed prior to addressing the hypothesis testing. For the first set of hypotheses, one sample T-Tests were performed to test if the sample means on the study variables differed from the scale mid-point. Two moderation models were performed for the second group of hypotheses, using the process macro developed by Hayes (2012) for SPSS. The conditional effect of parental exhaustion on relationship satisfaction and the frequency of conflicts was tested at values of dyadic coping (moderator) of mean plus/minus one standard deviation, controlling the effects of the number of children, their averaged age, number of days in telework, partner’s working status as telework, financial situation satisfaction and gender. All assumptions for these analyses were verified. A power analysis revealed that our sample size grants adequate power (0.95) to detect even a small effect size (0.10), as a minimum of 158 participants was needed.

## Results

### Describing the experience of being in lockdown

Descriptive statistics (Table 2) and one sample’s t-test were performed to understand the sample’s lockdown experience. Results showed that conflict frequency levels were not different from the middle point of the scale, which translates to medium scores for frequency of conflict (*t*(210) = − 1.62, *p* = .106, *d* = − 0.11). Scores for relationship satisfaction were found to be significantly higher than the medium scale point (*t*(208) = 12,3, *p* < .001, *d* = 0.86), meaning participants were satisfied with their relationships, consistent with interpretations for scores higher than the scale’s reference value of 3.5 (Hendrick et al., 1998). In terms of parental exhaustion, the sample revealed scores significantly lower than the medium point (*t*(208) = − 8.76, *p* < .001, *d* = − 0.60), while levels of dyadic coping were found to be higher than the mean point of the scale (*t*(208) = 8.56, *p* < .001, *d* = 0.60).

All study variables were significantly correlated (Table 2). Relationship satisfaction was negatively correlated with conflict frequency and parental exhaustion, and as conflict frequency increased, so did parental exhaustion. Dyadic coping showed significant correlations with all variables, associating with increased relationship satisfaction and decreased conflict frequency and parental exhaustion.

Significant correlations were also found between the study specific variables and sociodemographic variables (Table 3), showing that parental exhaustion decreased with longer relationships and cohabitations and as the youngest child’s age increased. Likewise, there was a decrease in this exhaustion when partners were in telework. As participants spent more days in telework, parental exhaustion levels were shown to increase, and the satisfaction with the relationship and dyadic coping were lower. Gender was also found to correlate significantly with parental exhaustion, demonstrating a prevalence of this stress for women.

**Table 3:** Correlations between Study and Sociodemographic Variables (n = 210)

Variables	Parental Exhaustion	Relationship Satisfaction	Conflict Frequency	Dyadic Coping
Relationship duration	− 0.26***	0.08	− 0.07	0.05
Cohabitation duration	− 0.28***	0.05	− 0.04	0.05
Number of children	− 0.05	0.01	− 0.07	0.03
Youngest child’s age	− 0.37***	0.04	− 0.08	0.02
Partner in telework^a b^	− 0.21**	0.11	− 0.08	− 0.12
Days in telework (n = 208)	0.14*	− 0.12*	− 0.01	− 0.16*
Satisfaction with the financial situation	− 0.14	0.24**	− 0.10	0.19**
Gender^a c^	0.16*	− 0.07	− 0.05	0.10

**Note**. ^a^Spearman’s correlation; ^b^0 = not teleworking partner; 1 = teleworking partner; ^c^0 = male; 1 = female

* *p* < .05; ** *p* < .01; *** *p* < .001

### Lockdown stress for cohabiting couples

Two moderation models recurring to the Process macro were used to test the predicting effect of parental exhaustion in relationship satisfaction and relationship conflicts and the moderation role of dyadic coping in each of these links while controlling for the role of covariates.

In the first model, results showed that predictors explained around 60% of relationship satisfaction (*R*^*2*^ = 0.60, *F*(9,190) = 30.9, *p* < .001). Parental exhaustion predicted a decrease in relationship satisfaction (*β* = − 0.11, *p* = .002), and dyadic coping predicted an increase in the same variable (*β* = 0.81, *p* < .001). Days in telework (*β* = 0.02, *p* = .573), partner in telework (*β* = − 0.01, *p* = .929), satisfaction with the financial situation (*β* = 0.03, *p* = .126), number of children (*β* = 0.00, *p* = .952), youngest child’s age (*β* = − 0.01, *p* = .425) and gender (*β* = 0.06, *p* = .644) did not predict the dependent variable. Moreover, a significant effect for the dyadic coping moderation was not found (*β* = 0.06, *p* = .149).

**Fig. 1:**
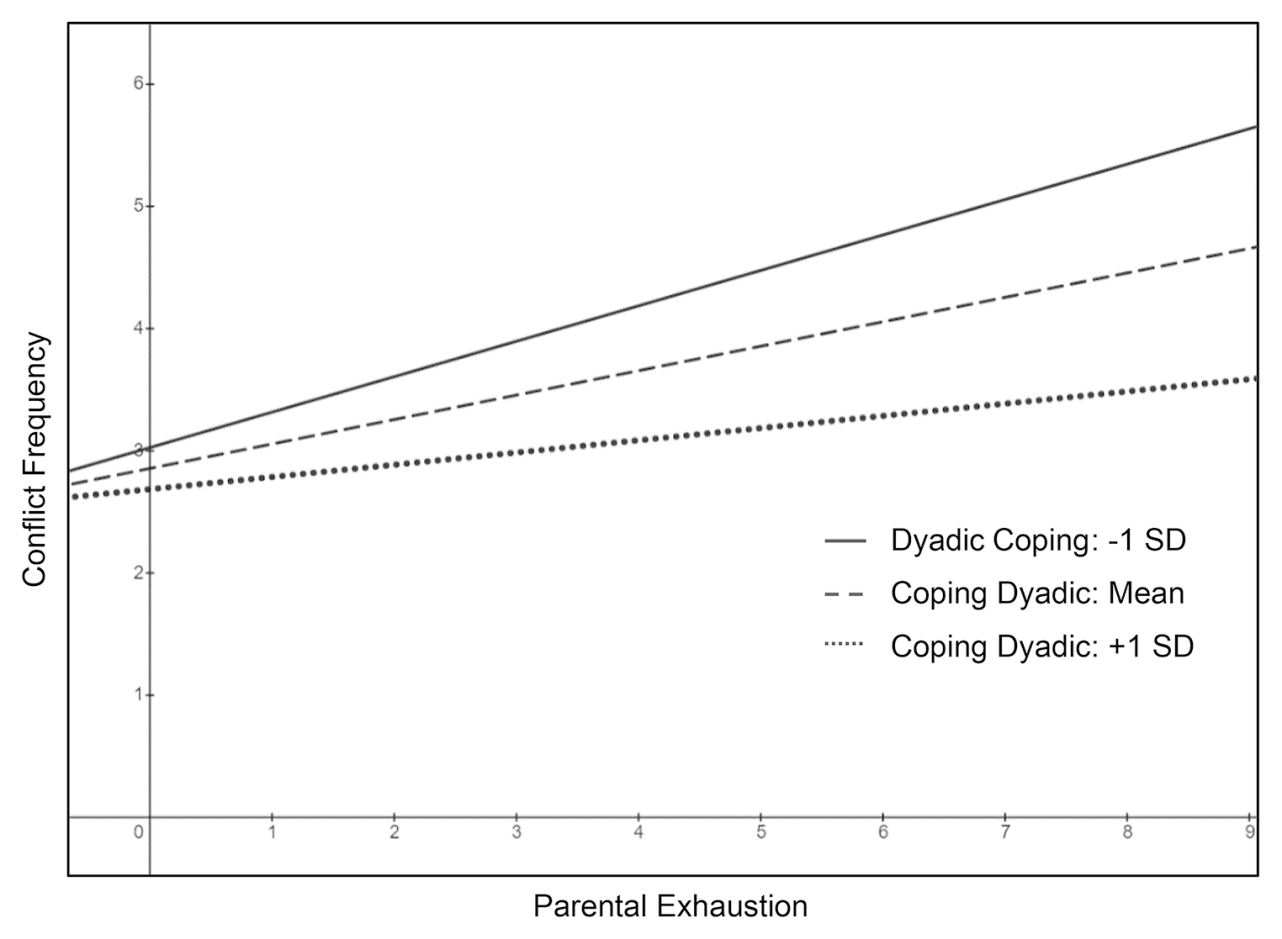
Dyadic Coping Moderating the Effect of Parental Exhaustion on Conflict Frequency

The second model revealed a predicting effect of around 19% of conflict frequency (*R*^*2*^ = 0.19, *F*(9,190) = 4.86, *p* < .001). Parental exhaustion predicted an increase in the perception of these conflicts (*β* = 0.20, *p* < .001) and dyadic coping a decrease (*β* = − 0.22, *p* = .006). None of the covariates were found to predict conflict frequency significantly: days in telework (*β* = − 0.09, *p* = .099), partner in telework (*β* = − 0.07, *p* = .594), satisfaction with the financial situation (*β* = − 0.00, *p* = .889), number of children (*β* = − 0.09, *p* = .333), youngest child’s age (*β* = 0.01, *p* = .424) and gender (*β* = − 0.24, *p* = .129). This time, a moderation effect was found (*β* = − 0.13, *p* = .023). Dyadic coping was found to moderate the effects of parental exhaustion on conflict frequency, as when it presents low (*β* = 0.29, *p* < .001) or mean scores (*β* = 0.20, *p* < .001), more conflicts are reported. By contrast, high dyadic coping scores did not buffer the effect of parental exhaustion on conflict frequency (*β* = 0.10, *p* = .146). Nevertheless, in the last case, the increment in conflicts caused by parental exhaustion is still lower when comparing with lower levels of dyadic coping (see Fig. 1).

## Discussion

The COVID-19 pandemic and its associated restrictions have been found to negatively impact couples’ dynamics by increasing external stress on the relationships (Ogan et al., 2021) and raising parenting demands in nature and intensity (Aguiar et al., 2021; Ares et al., 2021; Coyne et al., 2020; Mantovani et al., 2021). These changes are known to harm romantic relationships (Berryhill et al., 2016; Khajehei, 2016) and have been done it in the context of COVID-19 (Balzarini et al., 2020; Lee et al., 2021; Luetke et al., 2020; Ogan et al., 2021). As in other situations, we expected that dyadic responses to these stressors could buffer those negative effects (Bodenmann, 2005, 2006; Falconier et al., 2015). This study aimed to describe how couples with children experienced COVID-19 lockdown and to investigate how its associated constraints, namely exhaustion related to the parenting role, influenced the quality of their romantic relationships. We also intended to analyze how couples’ resources, such as dyadic coping, could mitigate possible adverse effects of these stressors on the relationship.

Results showed couple relationships as being characterized by medium levels of conflict frequency and above-average levels of relationship satisfaction. This contradicted our expectations and other evidence of relationship deterioration during the pandemic (Balzarini et al., 2020; Lee et al., 2021; Luetke et al., 2020). This may be because our sample was a convenience sample; therefore, couples who struggled the most may have self-excluded from participating in the survey. Another explanation may relate to the stability of these couples, as their relationships have an average duration of 18 years. However, the presence of high levels of dyadic coping in participants may represent a protecting factor against this decrease in relationship quality, as couples with intern resources to face stress against the relationship like dyadic coping end up revealing higher levels of relationship satisfaction (Balzarini et al., 2020; Pietromonaco & Overall, 2020) and lower frequency of conflicts (Lee et al., 2021). Robust positive correlations between dyadic coping and relationship satisfaction and strong negative correlations between dyadic coping and conflict frequency in this study support this argument. Moreover, while in lockdown, couples may have experienced fluctuation in the quality of their relationships (Vowels et al., 2021), so when asked about it, they may have averaged their experience. A resembling finding was shown in terms of parental exhaustion, as our participants revealed low levels of this parental burnout dimension, which goes against previous findings regarding parental burnout during COVID-19 (Aguiar et al., 2021; Marchetti et al., 2020), but can also be explained by the presence of these intern resources like dyadic coping (Skjerdingstad et al., 2021), something also supported by the strong negative correlations found. Again, Vowels and colleagues (2021) demonstrated how couples with children experienced many changes, including more cooperation and division of domestic and childcare tasks. This could also be the case here and explain these low stress levels related to the parental role. Characteristics of this sample may also explain some of the encountered results. As our participants were primarily women who are usually the principal caregivers and the main ones responsible for domestic labor, and there was a correlation between gender and parental exhaustion, low levels of this stress may be related to them being more used to the role and its demands. During the lockdown, fathers tended to feel a higher increase in parenting and domestic demands, probably due to this gender role differences (Carlson et al., 2022; Craig & Churchill, 2021). In addition, as our sample was highly educated and a significant correlation was found between satisfaction with financial satisfaction and relationship satisfaction, socioeconomic status and educational resources could constitute one privilege towards constraints associated with the pandemic, protecting their relationships from financial distress (Barton & Bryant, 2016).

When it comes to our second hypothesis, it was fully confirmed as parental exhaustion was found to predict a decrease in relationship satisfaction, even when controlling for family (e.g., number and age of children) and work-related (number of teleworking days, partner being in telework, satisfaction with the financial satisfaction) covariates, including gender. Parental exhaustion also explained an increase in conflicts between the couple. All this together aligns with previous research regarding the impact of parental stress on couple relationships (Berryhill et al., 2016; Lavee et al., 1996) and shows how in the context of the COVID-19 pandemic, couples’ perceptions of being drained away from resources due to taking care of their children were less satisfied with their relationship and had more disagreements.

Lastly, we partially confirmed our third hypothesis regarding the moderator effect of dyadic coping. We did not find dyadic coping moderating the impact of parental exhaustion on relationship satisfaction but only moderating the impact on conflict frequency. Indeed, the moderation effects of dyadic coping have been inconsistent. However, our findings may be seen as aligning with Lee and collaborators (2021), who also tested the moderating effect of dyadic coping on conflict frequency regarding pandemic-related stress and only found this buffering role on an earlier stage of the lockdown experience. This suggests what has been stated before that couples with more resources within the relationship and with more emotional and practical support from one another suffer less damage in their relationships (Falconier et al., 2015) and, in this case, end up fighting less when exhausted with their parental role. Since dyadic coping only buffers conflict frequency and not relationship satisfaction, this can be linked to the fact that relationship satisfaction is a broader construct, including different dimensions of the relationship. In contrast, conflict relates to communication behaviors mostly. As dyadic coping represents functional forms of communication, the path for a moderation effect may be more explicit. Also, conflict is a more acute dimension at the earlier stages of the pandemic; the impact of parental exhaustion over relationship satisfaction may occur when and if the strains associated with the pandemic are prolonged. As relationship satisfaction comprises a more general appraisal of the relationship, this dimension may be more protected from acute stressors.

### Implications, strengths, and limitations

This study represents an important contribution to comprehend the impact of the COVID-19 pandemic and its related restrictions on couples’ lives and dynamics, namely their conjoint adaptation towards stress involving taking care of their children and how that affects their romantic relationships. Moreover, it provides empirical evidence for Bodenman’s Systemic-Transactional Model (1995, 1997), specifically when it comes to the pandemic context, as it shows how couples with more intern resources, who perceive their partners as more supportive and use dyadic strategies to face stress are more prepared and suffer fewer negative consequences towards their relationship, than the ones who resort less to these dyadic responses. By investing in developing these coping competencies like communication and mutual support, couples could be more prepared to face external adversities and protect their relationship from increasing disagreements, and lower the general quality of their interactions (Randall & Bodenmann, 2009). Finally, this study represents important evidence for understanding how the parental role has individual and dyadic implications, namely on the quality of the romantic relationship. These results underline the pertinence of investing more in support for parents by providing easier access to practical help such as childcare services (Plantenga et al., 2009) and promoting interpersonal skills for better parenting cooperation, benefiting the whole family system (Feinberg, 2002).

It is possible to highlight some contributions of the present study. It constitutes one of the few studies developed during the early stages of the COVID-19 pandemic. Data was collected in the first months of the Portuguese lockdown, making it possible to have an in-depth knowledge of the families’ adaptation processes and experiences of those routine alterations. To our knowledge, few studies were built in such a short time and were able to capture these first impressions. Additionally, the present investigation focused on searching for associations not so deeply studied so far, like the impact of parenting-related exhaustion on the quality of the relationship in different dimensions, such as satisfaction and conflict. Also, the complementarity of evaluating two different variables to assess the quality of the relationship may be seen as a strength of our study.

Notwithstanding its contributions, our work includes important limitations. The study lacked the longitudinal component: by extending data collection pre-lockdown and allowing a comparison of relationships and stress before and after COVID-19, or during the several months and waves of lockdown that followed, it could have enabled us to make more grounded conclusions on the impact of the pandemic on couple relationships. The lack of sample representativity is also a limitation, as our sample mainly comprises women and participants in a stable socioeconomic situation. Thus, our data may not reflect the experiences of less socio-economic resourceful families or families with unique demands deriving from children’s needs. Moreover, balancing work and parental care is a gendered issue, and our study did not allow us to disentangle gendered experiences in this regard. Other investigations can benefit from a broader diversity in participants or, in a case, ponderation in terms of gender.

## Data Availability

The datasets generated during and/or analysed during the current study are available from the corresponding author on reasonable request.
